# Treatment of *Escherichia coli* contaminated water with different pulse-powered NTP configurations and analysis for post treatment efficacy

**DOI:** 10.1038/s41598-022-24248-9

**Published:** 2022-11-27

**Authors:** Sai Pavan Kalakonda, Gandluri Parameswarreddy, Emil Ninan Skariah, Benu George, T. V. Suchithra, T. K. Sindhu

**Affiliations:** 1 Electrical Engineering Department, National Institute of Technology Calicut, Calicut, Kerala 673601 India; 2 School of Biotechnology, National Institute of Technology Calicut, Calicut, Kerala 673601 India

**Keywords:** Microbiology, Environmental sciences, Engineering

## Abstract

A cost-effective and energy efficient method for water sterilization is a challenging demand in the present scenario where scarcity of pure water is rising. Non-Thermal Plasma (NTP) finds promising applications in environmental processes and has advantages over conventional water treatment methods. *Escherichia coli* contaminated water treatment using multiple pin plasma reactor and atmospheric pressure plasma jet reactor was undertaken in this work. High voltage pulsed power was used for generating non-thermal plasma in these reactors and various configurations were tested for treating the contaminated water. The most feasible configuration among these was identified from the treatment efficiency and survival rate plots of *E. coli* colonies. It was observed that with an exposure of 15 min NTP, 100 percent bacterial removal was achieved using plasma jet reactor configuration. The presence of bacteria after NTP-treated time was also checked and confirmed for complete removal of bacteria. An optimum time of 15 min plasma exposure for 100 ml was found to be effective for complete removal of microorganisms and the sterility was maintained up to 60 min after the treatment. Non-thermal plasma-based treatment of bacteria-contaminated water is found to be promising and could be considered for scale-up and analysis.

## Introduction

Access to pure water is a human right, and as stated by the United Nations (UN) in Human Rights to Water and Sanitation, ‘it entitles every individual to have access to sufficient, safe, acceptable, physically accessible and affordable water for personal and domestic use’. The UN defines ’safe’ as, free from microorganisms, chemical substances and radiological hazards that constitute a threat to a person’s health. There are conventional ways in which the water can be made free from microorganisms by using chlorine, ozone, chlorine dioxide, chloramines, and peracetic acid^1^. Despite the antimicrobial effect of the chemical methods, they tend to create by-products which are toxic in the long run^2^.

**Figure 1:**
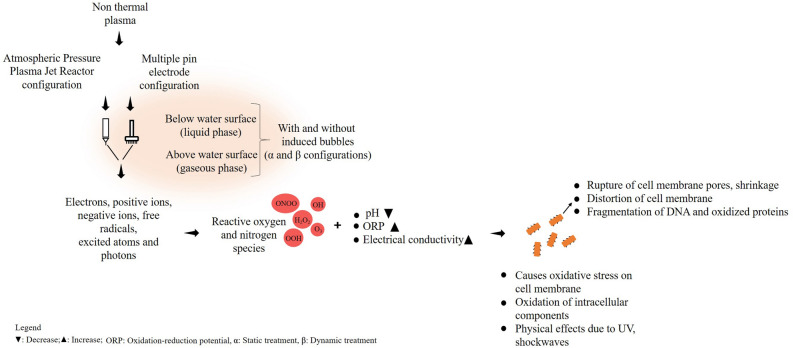
Basic schematic diagram representing the degradation mechanisms occurring due to NTP.

A ’safe’ way to disinfect water is using the technology of non-thermal plasma (NTP) which has an appreciable disinfecting ability without generating toxic by-products^3^. A survey reveals that the research on NTP sterilization systems has considerably increased over the past few decades^4,5^. NTP is an ionized gas consisting of reactive species and charged particles that operates at ambient temperature and pressure. The intrinsic antimicrobial properties of the reactive species make the NTP a better tool for viral and bactericidal sterilizations^6^. The chemical and antimicrobial properties of plasma activated water is well documented^7–11^ along with the conventionally used NTP reactor configurations. The basic process of sterilization (Fig. 1) using NTP above the water surface or below, leads to ionization reactions which causes chemical reactions in the gaseous and liquid phases. Thus, generation of reactive oxygen and nitrogen species (RONS), O$_{3}$, H$_{2}$O$_{2}$, ONOOH, HNO$_{2}$, HNO$_{3}$, superoxide radicals, perhydroxyl radicals etc. occurs which possesses antimicrobial properties. The pH, Oxidation-Reduction Potential (ORP) and electrical conductivity of the NTP treated water is thus altered favouring physical stress on the cell membrane of the microorganism. These factors, along with the associated UV radiation, photons and shock waves aids in the sterilization process. Sterilization is achieved by any or combination of process like cell membrane rupture, cracks and pores on the cell membrane, oxidation of intracellular components etc.

The application of a high electric field and ionizing the gas is the basic method of generating NTP and the sources that can be used for this purpose are high voltage-AC,-DC, and -pulses^12–14^. The high voltage pulsed power source generate short pulses that can impart larger energy to the NTP reactors in short time without causing gas heating and thereby increasing the efficiency of the intended NTP generation^15^. NTP can be generated in many forms like corona discharge, dielectric barrier discharge(DBD) or electron beam with a high voltage pulsed power source which facilitates better plasma characteristics^16,17^. The corona reactor/DBD reactor based NTP treatment with different configurations finds applications in water sterilization^3,18^.

Configurations with contact and contact-less discharge types were studied in previous literature for improving the water treatment efficiency^8,17^. Pin electrode configuration in such reactors enhanced the electric field around the tip thereby ionizing the air surrounding the pin to generate NTP with high intensity. A multiple pin HV electrode with seven tungsten needles powered by a HV pulsed power source was used for degradation of chlorobenzene using NTP and the study revealed that OH radicals played a major role in the detoxification of the solution^19^. A study on NTP generation by hollow electrode configuration through which the working gas was bubbled to the liquid for inactivation of *Staphylococcus aureus* has shown the presence of reactive oxygen and nitrogen species (RONS) in gas-liquid and liquid phase^20^. The investigation on inactivation of *E. coli* in water using NTP with air-argon mixture as working gas revealed that the immersed electrode configuration had more operational advantages than the one with HV electrode above water surface as the reactive species are directly generated in water^21^. NTP, which utilizes RONS generated has been a feasible process for clean water in many applications where conventional water treatment techniques cannot be used. Also NTP can be used in cascading with novel filtering techniques like CNT membranes^22^ for better and speedy removal of microbes in industrial applications. A comprehensive study on different non thermal plasma water sterilization methods and its effectiveness is a demand for development of future water treatment techniques.

The main objective of this study was to determine the time of bacterial inactivation in multidrug resistant *E. coli* contaminated water using different NTP reactor configurations and thereby determining the optimal electrode configuration. This optimal electrode configuration was further used for analyzing the sterility of plasma treated water samples after specific time periods. The survival rate curves were to be plotted for understanding the performance of different reactor configurations in static and dynamic water treatment schemes.

## Materials and methods

### High voltage pulsed power supply

A laboratory-developed variable HV pulsed power supply having a DC-DC flyback converter and modified Rotary Spark Gap (RSG) was used as the power source for NTP reactors^23^. The DC-DC flyback converter converts 12 V DC to high voltage DC which was then turned to pulses using the RSG. A 12 V, 32 Ah battery was used as the input source and the output voltage amplitude and pulse repetition rate can be varied. High voltage pulses of magnitude 15 kV and pulse repetition rate of 250 pps were applied to generate NTP in the reactors.

### NTP reactors

The NTP reactors used for static and dynamic water treatment systems in this work were multiple pin to plane electrode reactor and Atmospheric Pressure Plasma Jet Reactor (APPJR). The ground electrode for corona discharge in both the reactor arrangements was a 3 mm thick plane brass electrode of 60 mm diameter placed at the bottom of the 250 ml beaker containing the water sample. An air pump was used for APPJR to produce the plasma jet at the nozzle and also to induce bubbles in the contained water.

#### Multiple pin to plane electrode reactor configuration

**Figure 2:**
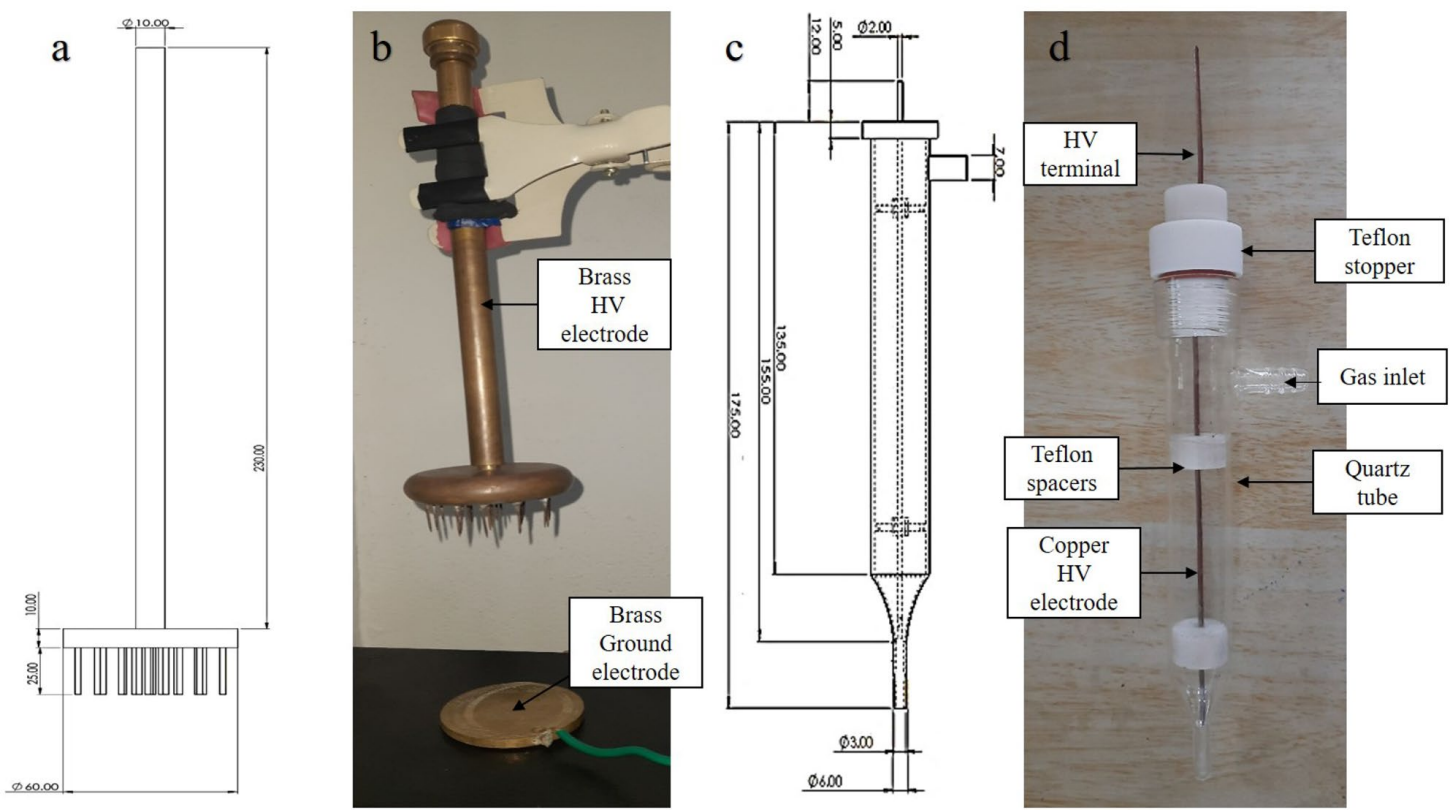
NTP reactor configurations (**a**) schematic cross-sectional diagram of multiple pin electrode with dimensions (in mm); (**b**) photograph of multiple pin electrode; (**c**) schematic cross-sectional diagram of APPJR with dimensions (in mm) and (**d**) photograph of APPJR.

A multiple pin HV electrode to plane electrode configuration was used to treat water in a static system as well as in a system with bubble-induced water movement (dynamic water system). The HV electrode has 19 pins with a length of 25 mm spread uniformly over a brass plate of diameter 60 mm (Fig. 2a, b).

#### Atmospheric pressure plasma jet reactor

The APPJR was used to generate a corona discharge and part of the reactor with nozzle was immersed in the water to inject NTP into it. APPJR is a funnel-shaped cylindrical reactor with a pin type HV electrode to enhance the electric field at the tip of the electrode (Fig. 2c). The tube is fabricated with quartz (Fig. 2d) and used in the dynamic water treatment system with the plane ground electrode. The glass tube is tapered to keep a uniform flow of gas, providing a jet of plasma at the nozzle and to increase the intensity of reactive chemical species.

### Sample preparation

Multidrug-resistant *E. coli* cultures were provided by Microbiology and Bioprospecting lab, National Institute of Technology, Calicut, and cultures were activated into 150 ml nutrient broth with overnight incubation at 120 rpm, 34 °C. The mother culture was subsequently re-cultured using a loopful of culture for each experiment in 5 ml broth under the above-mentioned culturing conditions. Each optimization experiment was performed in $1.13\times 10^{9}$ CFU/ml, which was standardized using the serial dilution method. 100 ml of sample was made and used for sterilization experiments. The spread plate technique was used for counting the colonies in the samples. Control plates for each experiment were maintained at the same conditions and all the experiments were carried out under aseptic conditions.

### Water sterilization using NTP

#### Static water treatment

**Figure 3:**
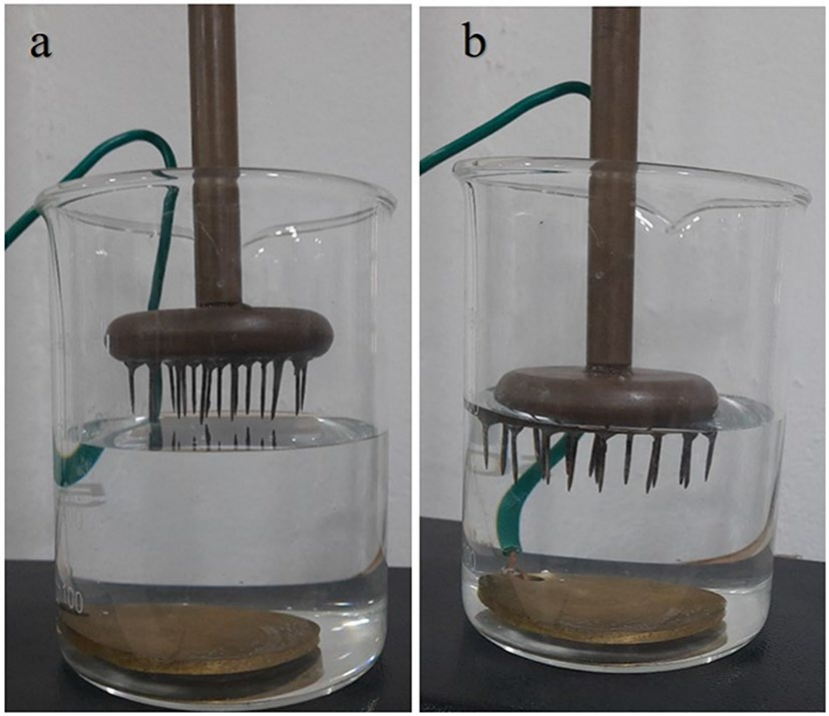
Static water treatment with multiple pin HV electrode to plane ground electrode in (**a**) $\alpha$-configuration and (**b**) $\beta$-configuration.

Multiple pin HV electrode was used for static water treatment using NTP. The treatment efficiency when the electrode placed above the surface of the water ($\alpha$-configuration) (Fig. 3a) and immersed in the water ($\beta$-configuration) (Fig. 3b) were observed over a period of time. Each experiment was performed by exposing the contaminated water to NTP for time periods of 10–30 min and 100 $\upmu$L of the sample was taken at every 5 min and spread plated on a nutrient agar petri dish. The colony count was observed for each time of treatment and corresponding survival rate curve was plotted.

#### Dynamic water treatment

**Figure 4:**
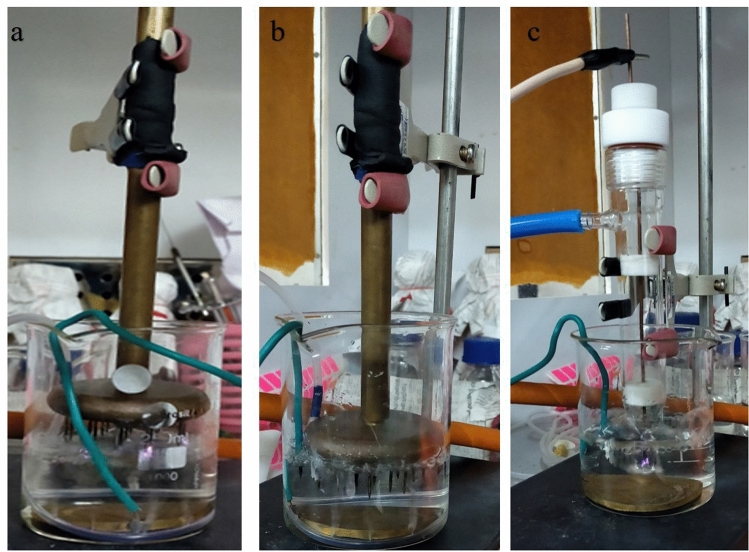
Dynamic water treatment with multiple pin HV electrode to plane ground electrode by arranging HV electrode in (**a**) $\alpha$-configuration and (**b**) $\beta$-configuration and with (**c**) immersed APPJR.

*(a) Multiple pin electrode configuration with bubble induced movement of water* An air tube with 6 holes distributed along the length was laid at the bottom circumference of the container to induce bubbles in the system. The performance of NTP based system for water treatment was observed in $\alpha$-configuration (Fig. 4a) and $\beta$-configuration (Fig. 4b). 100 $\mu$l of sample was taken at every 5 min over a period of 30 min starting from 10 min treatment and spread plated. The number of colony forming units were counted in each plate and survival rate curves were plotted.

*(b) Plasma jet reactor immersed in water* The APPJR was immersed into water (Fig. 4c) and the treatment efficiency was observed over a period of 30 min. Immersing APPJR into the water serves both the purpose of inducing the water movement by creating bubbles and injecting reactive species into it. The contaminated water was treated for 30 min and a 100 $\upmu$l of the sample was collected every 5 min starting from 10 min treatment to plate it on a nutrient agar plate using the spread plate technique. The number of colony forming units were counted on each plate and survival rate curve was plotted.

*(c) Effect of NTP exposure on water samples after the treatment period* It is imperative that the production of radicals and active chemical species in the water samples lead to concomitant reactions after the treatment. Thus the sterility of the treated sample is assumed to be maintained due to the presence of such reactions. To assess this phenomenon, analysis on the post treatment efficacy of NTP exposed water samples were done. The post treatment effect of NTP treated water was studied, using the APPJR reactor which showed better treatment characteristics. Different water samples were treated for 5 and 10 min, and their post treatment effectiveness was studied over a period of 60 min. Sample (100 $\upmu$l) was taken every 10 min and was plated on the nutrient agar medium. Based on the colony forming units observed at each treatment time, the survival rate curves were plotted.

## Results

All the experiments, sample preparation and spread plating were conducted inside a laminar air flow chamber to ensure aseptic conditions. Also, the experiments were done at atmospheric temperature and pressure. The multiple pin HV electrode in $\alpha$- and $\beta$-configurations for static and dynamic water systems and APPJR for dynamic water system were used to generate NTP and to treat multi-drug resistant *E. coli* contaminated water. Temperature of the water was observed during experiments using a thermal image camera (FLIR C3) and the values are given in Table 1. The mean values of temperature noted in each configuration were in the range 30$^0$C to 32$^0$C which indicates that no considerable heating action was taking place during the plasma treatment conforming to the non-thermal plasma operating conditions. An air pump with a flow rate of 4 lpm was used for inducing bubbles in the dynamic configurations and APPJR. The number of colonies present in water after NTP treatment were counted and compared with those of the control plate. The performance of reactors based on inactivation of multi-drug resistant *E. coli* being obtained, an optimum reactor was chosen and further tested for the post treatment effect in the treated water. The time required for complete sterilization of water was determined from the plots. The time periods upto which the sterility of NTP treated water samples remains intact was also determined from the corresponding experiments and its results.

**Table 1:** Mean value of water temperature measured during experiments.

S. no.	Type of configuration used	Temperature measured (°C)
1	Untreated water	30.2
1	Static water treatment $\alpha$-configuration	30.7
2	Static water treatment $\beta$-configuration	31.4
3	Dynamic water treatment $\alpha$-configuration	31.5
4	Dynamic water treatment $\beta$-configuration	31.6
5	APPJR immersed in water	31.8

### Static water treatment—time dependent analysis

**Figure 5:**
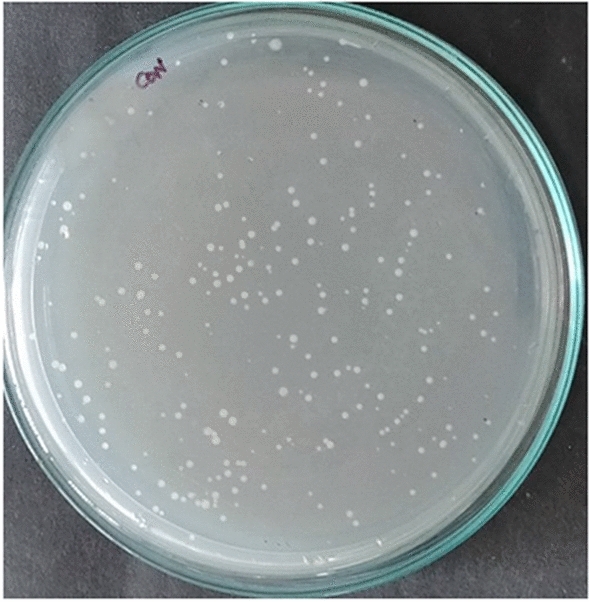
The control plate of untreated *E. coli* contaminated water for multiple pin HV electrode reactors.

**Figure 6:**
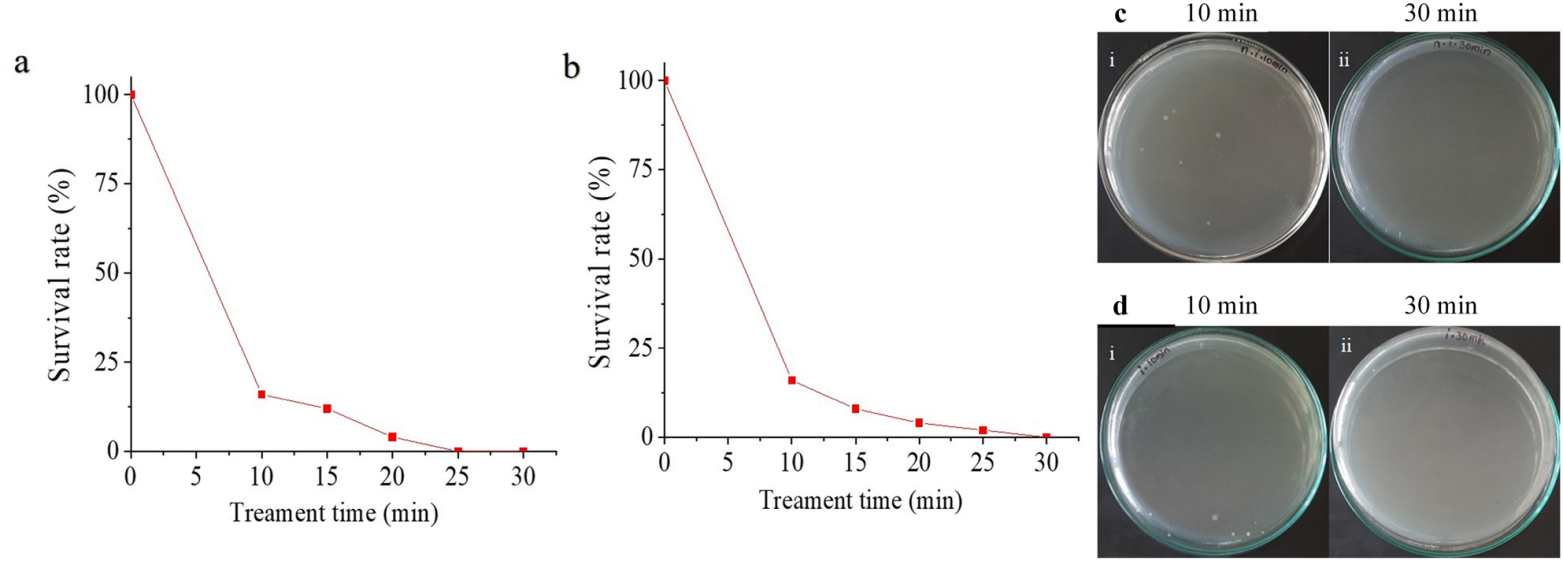
Static water treatment—percentage survival rate of *E. coli* against treatment time for multiple pin HV electrode (**a**) $\alpha$-configuration and (**b**) $\beta$-configuration; *E. Coli* colony in petri dish for different treatment time (**c**) $\alpha$-configuration, and (**d**) $\beta$-configuration.

The multi-drug resistant *E. coli* contaminated water was subjected to NTP in a static system using $\alpha$- and $\beta$-configurations of the multiple HV electrode. Control plates (Fig. 5) were also maintained and incubated for 24 h. The percentage survival rate was plotted against time of exposure/treatment time (Fig. 6a, b).

The treatment systems with multiple pin HV electrode in $\alpha$- and $\beta$-configurations exhibited a reduction in the colony count as the treatment time increased. An exposure for 15 min showed survival of *E. coli* as 22% in $\alpha$-configuration and 6% for $\beta$-configuration. 100% inactivation of *E. coli* was for 30 min treatment in $\alpha$- and $\beta$-configurations (Fig. 6c). $\beta$-configuration had better reduction compared to $\alpha$-configuration, due to the direct contact to generate reactive species in water and the immersed HV electrode avoided noise generated due to air discharge.

### Dynamic water treatment—time dependent analysis

*(a) Multiple pin electrode and bubble induced dynamic system*

**Figure 7:**
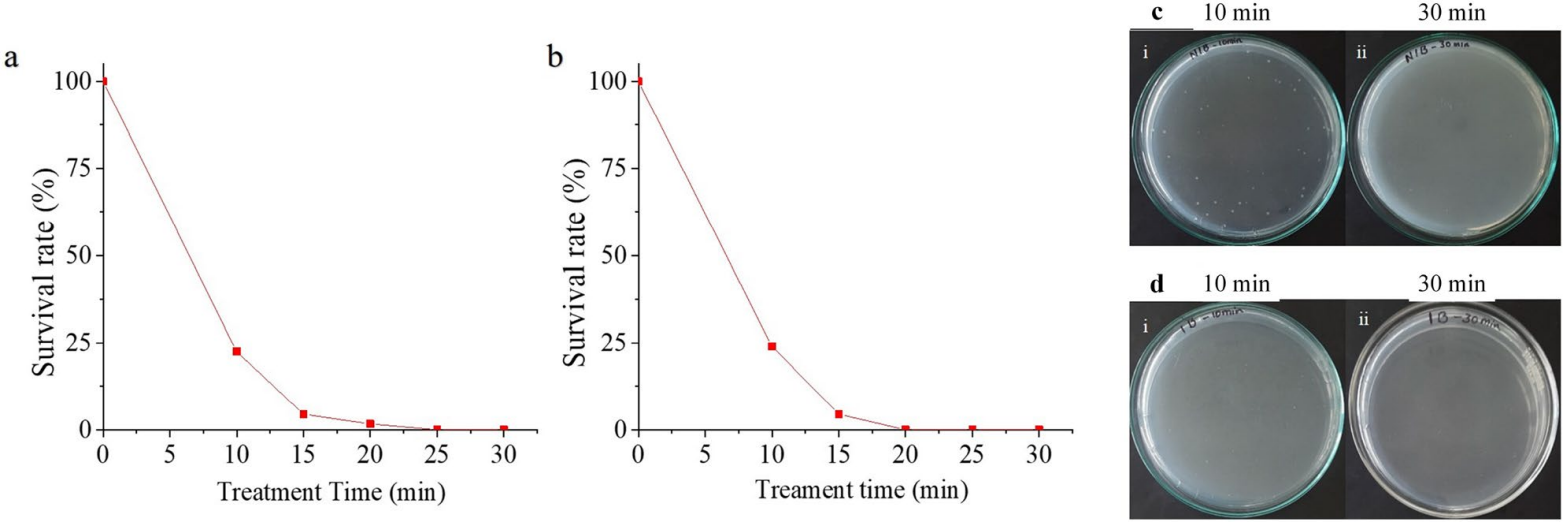
Bubble induced dynamic water treatment-percentage survival rate of *E. coli* against treatment time for multiple pin HV electrode (**a**) $\alpha$-configuration and (**b**) $\beta$-configuration; *E. coli* colony in petri dish for different treatment time (**c**) $\alpha$-configuration and (**d**) $\beta$-configuration.

The contaminated water was treated for 30 min and the percentage survival rate plotted for both $\alpha$-configuration and $\beta$-configuration with multiple pin HV electrode (Fig. 7a, b). The same control plate as before (Fig. 5) was maintained for this experiment. The survival of *E. coli* was 5% for 15 min of treatment and a 100% inactivation was achieved for an exposure of 20 min. It was observed from the spread plates (Fig. 7c) that the colony growth diminished with this configuration as the treatment time increased. Therefore, dynamic water treatment $\beta$-configuration was found to be better compared to static water treatment. Plasma was induced inside the liquid due to the air bubbles in this configuration rather than the surface plasma in $\alpha$-configuration. The higher efficiency was due to intensive production of radicals in the liquid, aided by the air bubbles.

*(b) APPJR immersed in water*

**Figure 8:**
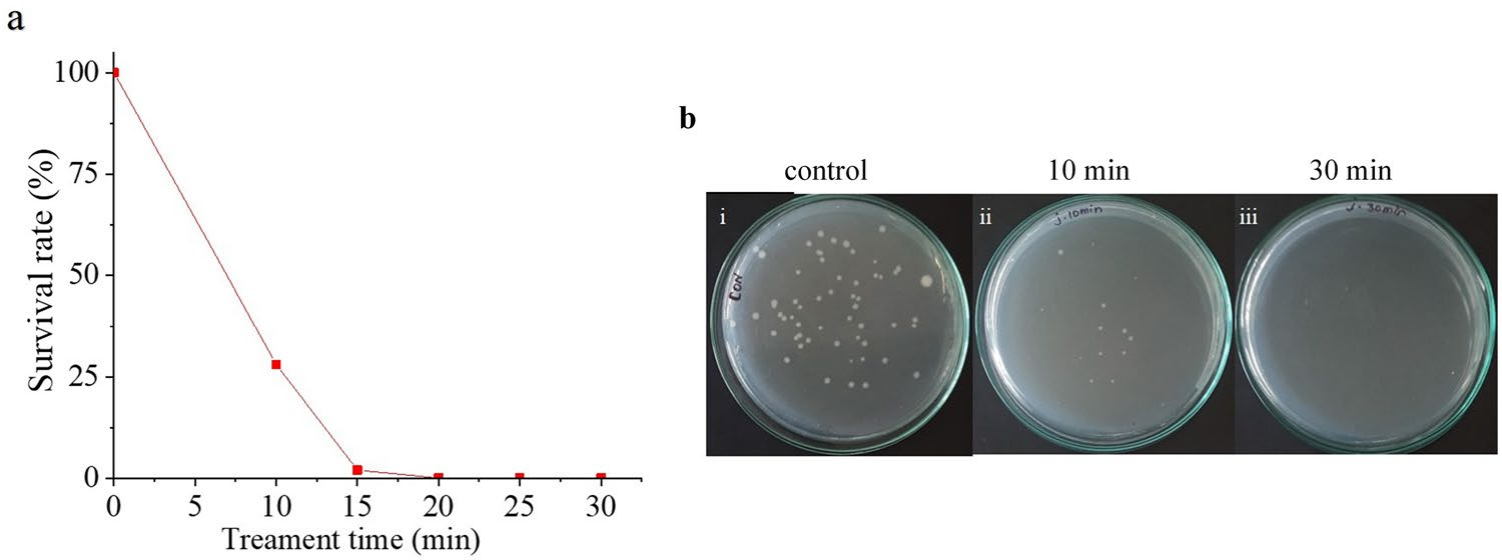
Dynamic water treatment with immersed APPJR (**a**) percentage survival rate of *E. coli* against treatment time; (**b**) *E. coli* colony in (i) control plate, and for treatment time (ii) 10 min and (iii) 30 min.

APPJR was used to treat contaminated water for 30 min and percentage survival rate was plotted against treatment time (Fig. 8a). In this scheme, the NTP produced by APPJR was injected into the water for different time periods. The control of untreated water was maintained and incubated for 24 h (Fig. 8.set b(i)). *E. coli* colony count was reduced to 100% within 15 min of treatment with APPJR system (Fig. 8.set b(ii, iii)). The sterilization efficiency and the time of NTP exposure needed for 100% removal of *E. coli* colonies was found to be better with this APPJR arrangement compared to the previous mentioned static and dynamic treatment systems. The plasma, emerging as a jet directly into the water along with the flow of forced air forms a positive environment for RONS production. The air bubbles introduced into the water from the jet has already undergone ionization and hence contains reactive species responsible for speedy disinfection/sterilization. This phenomenon reduced the effective treatment time compared to the other configurations. Therefore, APPJR system was determined as optimum reactor among all the configurations.

### Analysis of NTP exposed water samples after the treatment period

**Figure 9:**
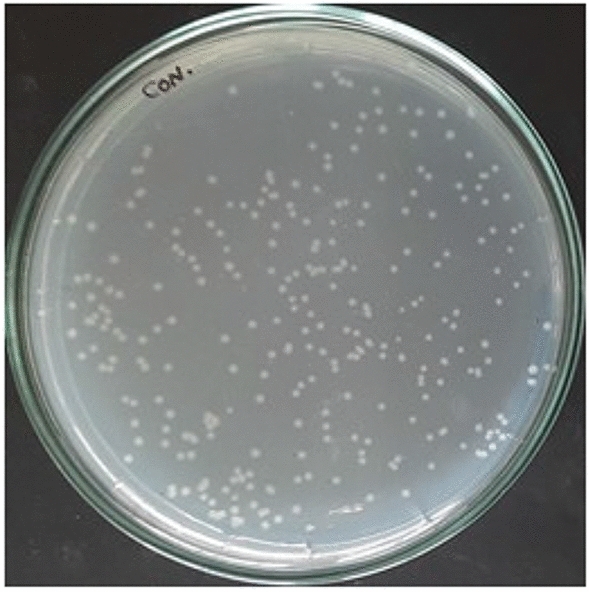
Control plate for post treatment analysis on APPJR treated water.

**Figure 10:**
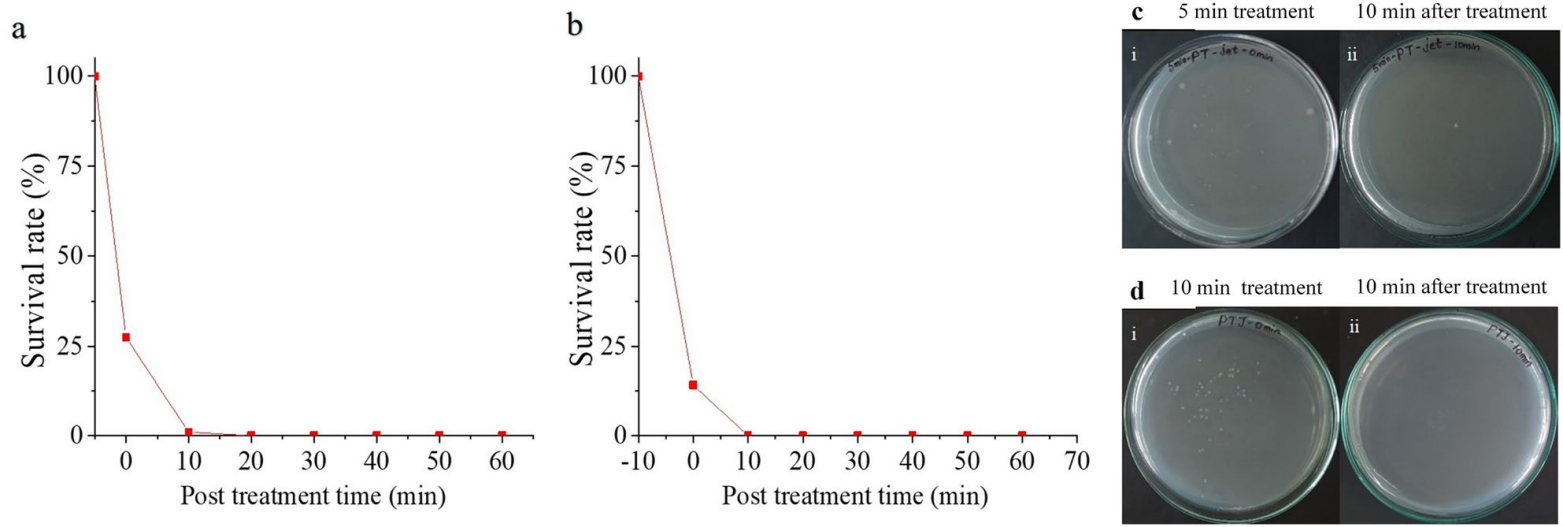
Post treatment analysis for APPJR treated samples-percentage survival rate till 60 min after treatment for (**a**) 5 min APPJR treatment and (**b**) 10 min APPJR treatment; (**c**) *E. coli* colony formed with 5 min treatment and 10 min after treatment; (**d**) *E. coli* colony formed with 10 min treatment and 10 min after treatment.

The presence of radicals or reactive species can possibly cause further reactions in the water after NTP exposure and retain the sterility of treated samples. This property was analysed by observing the colony growth in the samples taken at specific time periods after the treatment. Post-treatment analysis was done with the selected optimum reactor APPJR since it inactivated 100% of *E. coli* in water within 15 min of treatment compared with the other configurations. After treating the water with NTP from the APPJR, the water sample was kept idle in the container/beaker. Samples from this beaker were taken and spread plated at every 10 min till 60 min. These spread plates, along with the control of untreated contaminated water (Fig. 9) was incubated for 24 h. The colony development of microorganism in the treated water for different time intervals after NTP exposure were thus examined from these plates. The percentage survival rate calculated from the colony count was plotted against post treatment time (Fig. 10a, b).

The survival of *E. coli* in APPJR treated water after 5 min and 10 min was observed as 25% and 20% respectively. By 10 min of post treatment time, 100% of *E. coli* was inactivated for both the cases. The reactive species generated during NTP treatment were inactivating remaining *E. coli* bacteria during this time preventing further colony formation. Similar performance was observed for the samples kept for longer time, upto 60 min after the NTP exposure (Fig. 10c, d). Water treatment method using NTP was thus able to prevent further colony growth in the samples for a considerable time period, which is a desirable property. The 15 min NTP treatment time observed previously with APPJR can be assumed as the optimum for complete sterilization. This analysis was helpful to assess the efficacy of the process and to optimize the operating parameters. Therefore, 15 min of NTP treatment with APPJR was found as the optimum method for sterilizing 100 ml contaminated water which retained the survival rates till 60 min.

## Discussions and conclusions

Transmission of disease-causing microbes is possible through direct contact or by any medium. One of such and most crucial medium of transmission is water which causes 80% of all the diseases that are transmitted, according to World Health Organisation (WHO). It is estimated that around 3.1% of deaths in the world are caused due to unhygienic and polluted water. Water may contain bacteria like *E. coli* which causes diseases like cholera, dysentery, diarrhoea, polio and typhoid fever^24,25^. NTP has the capability to inactivate different bacteria and viruses, therefore can be used as an environmental friendly tool for virus and bacterial disinfection^6^.

The present investigation focuses on inactivation of multidrug resistant *E. coli* in static and dynamic water systems using a multiple pin HV electrode arranged in different configurations and an APPJR immersed in water. The multiple pin HV electrode was used in $\alpha$- and $\beta$-configurations in both static and dynamic water systems. A multiple pin electrode made of brass attached with 19 iron pins was used as HV electrode for the generation of NTP. This electrode was used in $\alpha$- and $\beta$-configuration and it was verified that $\beta$- configuration showed better results owing to the operational advantage of having both the electrodes in water. Immersed electrodes tends to improve the plasma process when generated in-liquid than the surface-generated plasma. It was observed that, with NTP treatment using multiple pin HV electrode, the colony count reduced as the treatment time was increased, the trend which was reported in previous literature^26,27^. For 15 min of treatment, the survival of *E. coli* was 22% in $\alpha$-configuration and 6% in $\beta$-configuration. Inactivation of *E. coli* was 100% for 30 min treatment with $\alpha$- and $\beta$-configurations. $\beta$- configuration exhibited better reduction compared to $\alpha$-configuration due to direct contact with water, thereby generating reactive species in water. These results were comparable with the previous works^28^ and the treatment time was reduced further in the present work. The treatment efficiency in static configurations can be attributed to reduction of pH of the water samples^28^.

For dynamic water treatment, the survival of *E. coli* was 5% for 15 min of treatment, and a 100% inactivation was achieved for exposure of 20 min. Compared to the previous studies by researchers^29–31^, the present study confirms better sterilization by providing bubbles in the water sample during NTP exposure. Therefore it is inferred that dynamic water treatment $\beta$-configuration is better compared to static water treatment. Inducing bubbles in the liquid causes the reactive species dispersion all over the water, increases the H$_{2}$O$_{2}$ and reduces the treatment time. In a similar way, for APPJR immersed in water, the *E. coli* colony count was reduced by 100% with 15 min of treatment. Reduction in *E. coli* colony count compared to previous water treatment methods with multiple pin electrode was found to be better in this case. The usage of air as the working gas and APPJR powered with high voltage pulses were the advantages gained along with satisfactory sterilization effects compared to the previous literature^32,33^. This is because of the radicals and active species generated inside the reactor, which is induced into and circulated throughout the entire volume of the water sample. Therefore, the APPJR system was determined as the optimum reactor among all the configurations. The present work investigated the effect five different NTP configurations on the sterilization of *E. coli* contaminated water samples. Satisfactory results were obtained and it was inferred that usage of bubbling inside water sample aided sterilization. The method can be extended for higher volume of water and multiple contaminants.

Analysis of the APPJR treated water sample was done after certain time period of NTP exposure. This was to assess the colony growth and hence the property of NTP treatment in retaining the sterility of water sample. This analysis was done for the water samples treated for 5 min and 10 min with APPJR. These samples, kept idle after the NTP exposure, were spread plated for 60 min at intervals of 10 min each. The percentage survival rate was plotted against post treatment time to assess the colony formation after the NTP treatment. The APPJR survival of *E. coli* for 5 min was 25% and for 10 min was 20% and after 10 min of NTP treatment, 100% of *E. coli* was inactivated for both the treatment times. It was observed that reactive species generated during treatment time were inactivating the remaining *E. coli* bacteria, thus preventing further colony multiplication. Therefore, 15 min of treatment time was found to be the optimum for sterilizing 100 ml contaminated water.

Water treatment systems can be enhanced with the non-thermal plasma based technique for attaining maximum sterilization effects. This work investigated different configurations of NTP based water treatment systems and their effect on *E. coli* contaminated water samples. It was inferred that NTP assisted with water bubbles enhance the antimicrobial process. Also NTP injected as a jet into the water sample showed superior results. The efficacy of treated water after plasma exposure was also analyzed to understand the time to which sterilization process continues even after the exposure. The quantitative analysis of parameters revealed potential scope and feasibility of NTP based water treatment schemes. The scope of treating multiple contaminants in a larger volume of water has to be tested and verified for commercial/industrial implementation.

## Data Availability

All data generated or analysed during this study are included in this published article.
